# Total skin electron therapy vertical profiles measured using radiochromic film

**DOI:** 10.1002/acm2.13941

**Published:** 2023-02-22

**Authors:** Nicole I. C. Cappelletto, Martin G. Shim

**Affiliations:** ^1^ School of Interdisciplinary Sciences McMaster University Hamilton Ontario Canada; ^2^ Department of Medical Physics Juravinski Cancer Centre, Hamilton Health Sciences Hamilton Ontario Canada

**Keywords:** gafchromic film, quality assurance, radiochromic film, total skin electron therapy

## Abstract

**Background:**

Vertical dose profiles of Total Skin Electron Therapy (TSET) electron fields are often measured using ionization chambers (ICs); however, resulting protocols are tedious and time consuming due to complex gantry arrangements, numerous point dose measurements and extra–cameral corrections. This inefficiency is reduced when using radiochromic film (RCF) dosimetry through simultaneous dose sampling and the elimination of IC‐related measurement corrections.

**Purpose:**

To investigate the feasibility of RCF dosimetry for TSET vertical profile measurements and establish a novel RCF based vertical profile quality assurance protocol.

**Methods:**

Thirty‐one vertical profiles were measured using GAFChromic^®^ EBT‐XD RCF on two matched linear accelerators (linacs) over 1.5 years. Absolute dose was quantified using a triple channel calibration method. Two IC profiles were collected for comparison to RCF profiles. Twenty‐one archived IC measured profiles from two different matched linacs from 2006 to 2011 were analyzed. Inter‐ and intra‐profile dose variability was compared between dosimeters. The time required for the RCF and IC protocols was compared.

**Results:**

RCF measured inter‐profile variability ranged from 0.66%–5.16% and 1.30%–3.86% for the two linacs. A 0.2%–5.4% inter‐profile variability was observed for archived IC measured profiles. RCF measured intra‐profile variability ranged from 10.0%–15.8%; six of 31 profiles exceeded the EORTC ± 10% limit. Archived IC measured profiles exhibited lower intra‐profile variability (4.5%–10.4%). RCF and IC measured profiles agreed in the center of the field; however, RCF doses measured 170–179 cm above the TSET treatment box base were ∼7% greater. Modification to the RCF phantom eliminated this discrepancy, resulting in comparable intra‐profile variability and agreeance with the ±10% limit. Measurement times were reduced from 3 h (IC protocol) to 30 min (RCF protocol).

**Conclusions:**

RCF dosimetry improves protocol efficiency. RCF has been established as a valuable dosimeter for TSET vertical profile quantification when compared to ICs as the gold standard.

## 1 INTRODUCTION

Total skin electron therapy (TSET) is a well‐established radiotherapeutic technique which uses large (∼200 × 80 cm^2^) electron fields to treat wide‐spread, superficial malignancies.^1^, ^2^ Of critical importance is the uniformity of the electron field, which is assessed during quality assurance (QA) via the acquisition of vertical dose profiles. Acceptable variations in dose distributions for vertical profiles are ±8% within the central 160 × 60 cm^2^ area of the treatment plane and/or ±10% of the prescribed dose as per AAPM TG‐30^1^ and the EORTC group,^3^ respectively.

Ionization chambers (ICs) are often used to measure vertical profile doses, however, resulting QA procedures are inefficient and impose a significant time burden on physics staff. This stems from the fact that a single IC can only sample one profile height at a time, with typically eight irradiations required per height to account for dual field configurations^4^ and extra–cameral corrections.^5^ Specifically, the IC must be irradiated twice for each gantry angle and polarity (±300 V). Sampling enough points to maintain sufficient profile resolution results in data collection times of approximately 3 h per profile.

Radiochromic film (RCF) is an alternative dosimeter which can increase the efficiency of the vertical profile QA protocol through simultaneous profile sampling and the elimination of IC‐related corrections. Previously, in‐house attempts using RCF to measure TSET profiles have seen limited success due to dose uncertainties measuring >3%. However, RCF dosimetry has greatly advanced since the AAPM TG‐55 report,^6^ and as a result, an updated AAPM TG‐235 report^7^ was published in 2020. RCF, specifically GAFChromic^®^ EBT‐XD RCF (Ashland Inc., NJ),^8^ is a desirable dosimeter as it is self‐developing, insensitive to visible light, and exhibits near water equivalence,^7^ low energy dependence,^8^, ^9^ and dose rate independence.^9^ Post irradiation, a triple channel calibration method is used to quantify absolute dose.^7^, ^10^ Unlike single and dual channel methods, the triple channel method allows for removal of the non‐dose‐dependent portion of a film image and provides greater sensitivity at lower doses when using the red color channel.^10^, ^11^ With dosimetric uncertainty reduced to <3% when using a triple channel calibration method,^12^ we thought it prudent to investigate if RCF may be used to measure TSET vertical profiles.

In this work, the feasibility and time efficiency of RCF dosimetry for TSET vertical profile quantification is assessed by comparing RCF and IC measured profiles and assessing the long‐term reproducibility of RCF measurements over 1.5 years.

## 2 MATERIAL AND METHODS

**FIGURE 1:**
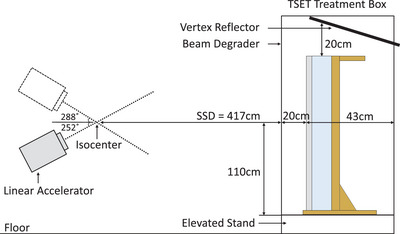
TSET linac, treatment box and phantom setup. Film and IC vertical profile setup is illustrated with the beam angles and relevant dimensions.

Two matched Varian TrueBeam^®^ linacs (Varian Medical Systems, CA), referred to as Linac 1 and 2, equipped with a 6MeV high dose rate total skin electron (HDTSe^−^) mode were used to generate 3–5MeV electron fields at a source‐to‐surface distance (SSD) of 417 cm (Figure 1).^13^ The dual‐field modified Stanford technique was used with gantry angles of ±18˚ from the horizontal.^4^ The multi‐leaf collimators were fully retracted and the collimator angle and field size were set to 0° and 40 × 40 cm^2^ at isocenter, respectively, producing a useable 200 × 80 cm^2^ field at a SSD = 417 cm. All TSET RCF vertical profiles were measured at d = 6.0 mm and 12.0 mm, with and without a 6 mm thick Lexan™ beam degrader, respectively. These depths correspond to d_max_ in water for the 6MeV extended SSD beams.

### 2.1 Dosimeter and phantom preparation

**FIGURE 2:**
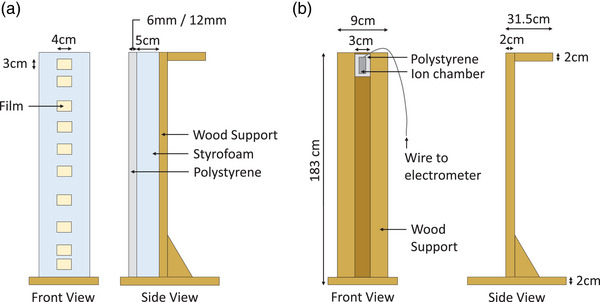
RCF and IC phantoms. Phantom design and dosimeter set up for (a) RCF and (b) IC. Two polystyrene attachments with different thicknesses (i.e., 6 mm vs. 12 mm) were used in the RCF phantom set up, depending on the beam degrader configuration (i.e., with or without, respectively). Similarly, two different thicknesses of polystyrene were used for the IC holder.

A 185 cm tall phantom composed of a wooden vertical support with Styrofoam and polystyrene attachments was developed in‐house for use with both the RCF and IC (Figure 2).

*RCF*—GAFChromic^®^ EBT‐XD RCF (Ashland Inc., NJ) was used for film dosimetry. Eleven 3 × 4 cm^2^ pieces of film were used per vertical profile, keeping their landscape orientation consistent for scanning (Section 2.4).^8^, ^12^ One of the eleven films acted as a zero‐dose control while the remaining ten films were positioned on the front face of a 5 cm thick Styrofoam attachment at the following ten heights relative to the floor of the TSET treatment box: 4, 10, 30, 60, 90, 110, 130, 150, 170, and 179 cm (Figure 2a). Sheets of 6.0 mm and 12.0 mm thick polystyrene overlaid the film and were used as buildup with and without the 6 mm thick beam degrader, respectively. The polystyrene thicknesses were chosen to closely match d_max_ for water. The phantom was placed in the TSET treatment box, with the front surface of the buildup set orthogonal to the incident beam and the 110 cm height positioned 43 cm from the back of the treatment box (Figure 1). With the gantry angle set to 270°, the horizontal crosshair coincided with the profile normalization height at 110 cm and the vertical crosshair coincided with the film centers.

*IC*—The Exradin A1SL thimble IC (Standard Imaging Inc., WI) was inserted into a rectangular polystyrene casing to set the measurement depth to d_max_ in water with and without the degrader. With the IC axis vertical, the IC was positioned on the wooden support, one height at a time at a combination of seven of the following heights relative to the TSET treatment box: 4, 20, 30, 50, 60, 80, 90, 110, 120, 140, 150, 170, and 179 cm (Figure 2b). The IC was connected to an electrometer located outside of the treatment room via a coaxial cable that was run on the periphery of the treatment box to minimize exposure to the large electron beams. The phantom was placed in the TSET treatment box as described for the film. Additionally, 21 archived IC measured profiles (ten with beam degrader; 11 without beam degrader), measured on two matched Varian 2100EX linacs from 2006 to 2011, were analyzed for comparison to film. Both linacs were installed in nearly identical treatment rooms; the same methodology, phantoms, electronic hardware and irradiation plan were used to measure the archived profiles.

### 2.2 Film calibration

Film calibration was implemented using FilmQA Pro (v.5.0, Ashland Inc., NJ).^12^ Six films were irradiated with doses ranging from 0 to 6 Gy and an optical density *versus* dose curve was fit to a rational function given by Equation 1:

(1) $$X\left(D,n\right)=a+\;\frac{b}{\left(D-c\right)}$$

where *X(D,n)* is the film transmission of the n^th^ color channel, measured from film exposed to dose *D*, and *a*, *b* and *c* are fit constants. Each film lot was calibrated once. The film at height 110 cm was used for linear calibration scaling to account for variations in linac output and scanner performance.

### 2.3 Irradiation

Both dosimeters were irradiated using 6MeV electrons in HDTSe^−^ mode, delivering a dose rate of 2500MU/min to the isocenter and 0.05cGy/MU to d_max_ at an extended SSD = 417 cm. The vertex reflector was raised to the maximum height for all profiles. This device is a 50 × 50 × 0.1 cm^3^ lead sheet angled at 30° to the horizon that is typically raised to 10–20 cm above a patient's head during treatment (Figure 1).^14^

*RCF—*Films were simultaneously irradiated with 10,000MU from each gantry angle to deliver 500cGy to the film at the 110 cm height. Following irradiation with both gantry angles, the films were stored in the dark and analyzed 24 h post‐irradiation.

*IC—*For each of the seven heights, the IC was irradiated with 1000MU four times—twice with an applied voltage of +300 V and twice with ‐300 V. The current was averaged to account for extra‐cameral effects. This was then repeated for the second beam angle, for a total of 8 irradiations per height.

### 2.4 Film dosimetry

Films were scanned using the Epson 11000XL scanner (San Jose, CA) in transmission mode, with 48‐bit color depth and 72dpi spatial resolution. Films for each profile were scanned simultaneously in landscape mode by centering them along the longitudinal axis of the scanner bed to decrease susceptibility to lateral response artifact.^15^ A 4 mm thick glass compression plate was placed over the films for scanning. All image correction features were turned off and the RGB image was saved as a TIFF file with no compression.

To calculate dose from film transmission, a 1 × 1 cm^2^ region of interest was drawn at the center of each film to first calculate mean transmission per height. The mean transmission for the zero dose and the 110 cm height films were used to linearly scale the calibration curve, effectively compensating for variations in linac output and scanner response. Triple channel uniformity correction was subsequently applied. Finally, the dose measured by each film was calculated using the red channel transmission data and the scaled calibration curve. Vertical profiles consisted of the measured dose at each height normalized as a percent of the dose delivered to 110 cm.

## 3 RESULTS AND DISCUSSION

Eight film measured profiles were acquired per linac and beam degrader configuration (i.e., with and without the beam degrader) over T = 532 days, where the first profile was collected on day T = 0. One profile was omitted (i.e., Linac 1 – without beam degrader, T = 0), resulting in a total of 31 useable film measured vertical profiles. Film measurement variability was quantified by comparing both inter‐ and intra‐profile dose ranges. Intra‐profile variability was calculated using the difference between the minimum and maximum normalized dose in a single profile, while inter‐profile variability was calculated as the difference between the minimum and maximum normalized dose between profiles, per height. A total of two IC measured profiles were measured on Linac 2 (i.e., with and without the beam degrader) and were used for qualitative comparison to film profiles taken 1 month apart. Otherwise, 21 archived IC measured profiles, measured on two matched Varian 2100EX linacs from 2006 to 2011, were used for comparison to film. Variation of ≤ ± 5% is required for comparable performance, relative to IC profiles.

**FIGURE 3:**
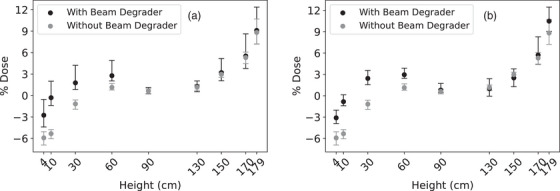
RCF inter‐profile variability. Mean relative dose as a function of height for (a) Linac 1 and (b) Linac 2, with and without the beam degrader. Error bars represent inter‐profile variability, measured as the minimum and maximum reported normalized dose.

Film measured inter‐profile dose variability as a function of height is summarized in Figure 3. Film measurements were stable, with relative dose ranges of 0.66%–5.16% and 1.30%–3.86% for Linac 1 and 2, respectively. Linac 1 without the beam degrader had the most consistent measurements compared to the other three configurations. Dose variability was height dependent, with increased variability observed at the edges of the field, notably, at heights 170 and 179 cm and, to a lesser extent, 4 cm. Archived IC profiles exhibited similar dose variability of 0.2%–5.4%, suggesting film provided similar measurement variability.

**FIGURE 4:**
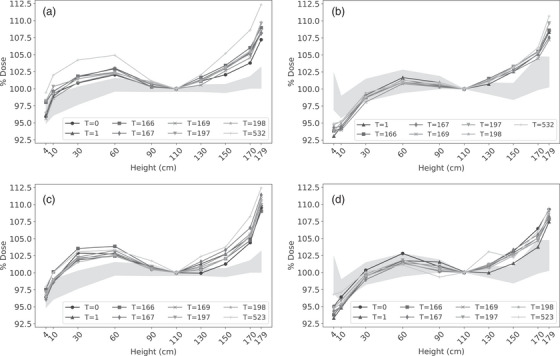
RCF intra‐profile variability. Vertical profiles measured with RCF on Linac 1 (a) with the beam degrader and (b) without the beam degrader and Linac 2 (c) with the beam degrader and (d) without the beam degrader. Doses are reported as a percent of the prescribed dose at 110 cm. Profiles are labeled by the day they were measured, starting with day T = 0. The shaded region represents the maximum and minimum percent dose reported at matched heights from ten and 11 archived IC measured profiles with and without the beam degrader configuration, respectively.

Intra‐profile dose ranges for the 31 film measured profiles varied between 10.0% and 15.8% (Figure 4). Although these were within the allowable 20% range, only 81% (25/31) of profiles exhibited doses within the acceptable dose variability of ±10%.^1^, ^3^ As with the inter‐profile analysis, Linac 1 (with beam degrader) consistently had lower intra‐profile dose ranges. In contrast, archived IC measured profiles exhibited a lower average dose variability of 4.5%–10.4% (shaded regions; Figure 4). A consistent discrepancy was noted at height 179 cm where film doses were approximately 7% greater compared to IC measurements. At height 4 cm there was good agreement for both linacs with the beam degrader, however, film doses were approximately 5% lower than IC with the degrader removed.

**FIGURE 5:**
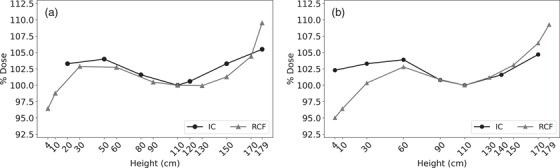
RCF versus IC profiles. Comparison of RCF and IC profiles measured 1 month apart on Linac 2 (a) with the beam degrader and (b) without the beam degrader. Doses are reported as a percent of the prescribed dose at 110 cm.

To account for dose variation over time, one film and IC profile were measured 1 month apart with and without the beam degrader (Figure 5). This confirmed the observations from Figure 4, which suggested good agreement in the central portion and discrepancies at the peripheries of the profiles. The latter were presumably due to the different scatter dose measured by each dosimeter where the backing of the film and IC were Styrofoam versus polystyrene, respectively.

**FIGURE 6:**
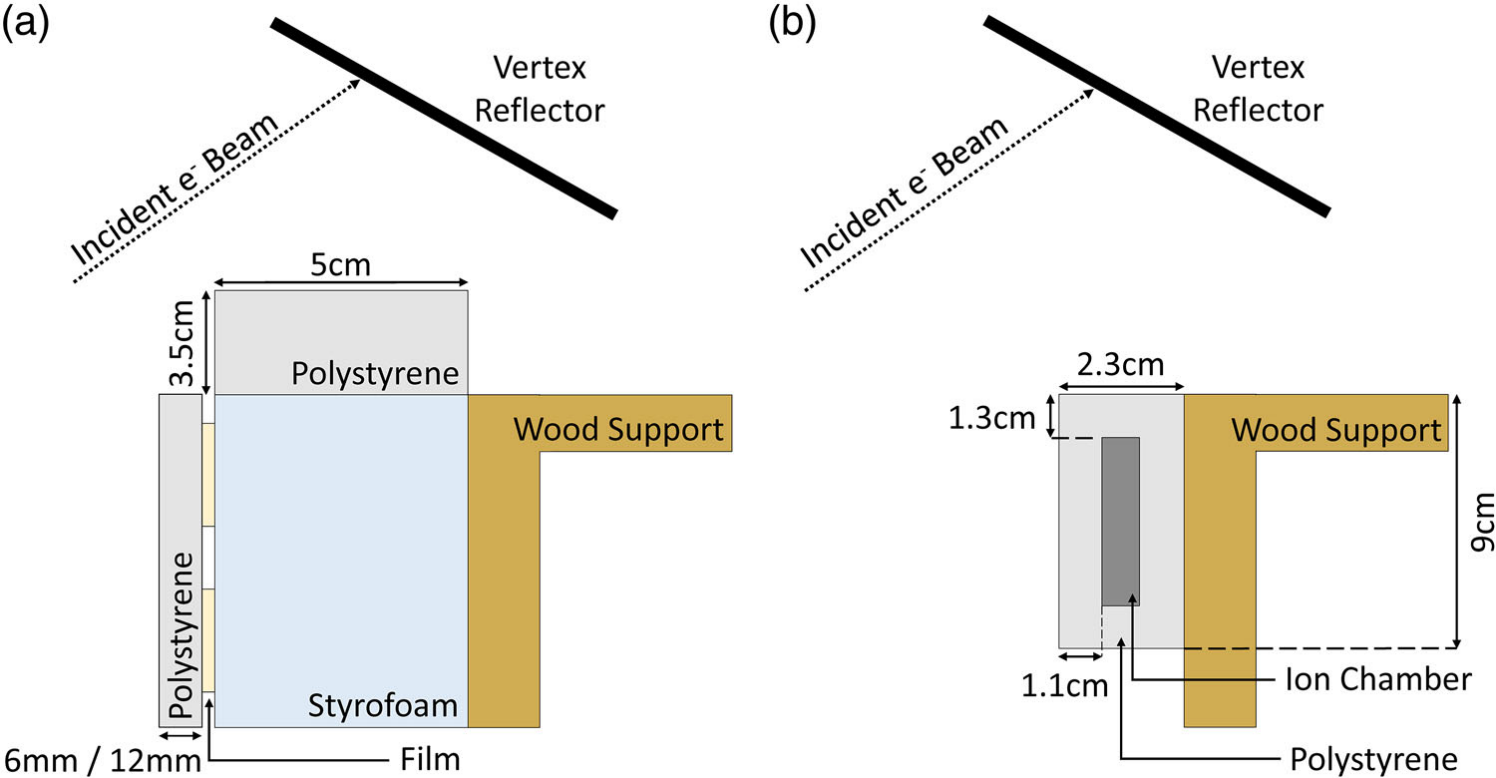
Updated RCF phantom. Side view of the top of (a) the RCF phantom and (b) the IC phantom, with the addition of a 3.5 cm thick slab of polystyrene to the top of the RCF phantom to enclose the RCF similarly to the IC.

**FIGURE 7:**
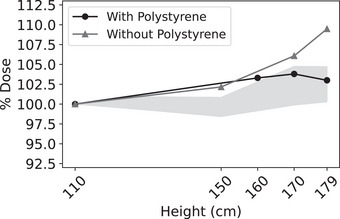
Updated phantom RCF intra‐profile variability. Top portion of RCF measured vertical profile measured with Linac 2 – without the beam degrader. Profiles are shown with and without the additional 3.5 cm thick piece of polystyrene (Figure 5). The shaded region represents the maximum and minimum percent dose reported at matched heights from 11 archived IC measured profiles without the beam degrader. Doses are reported as a percent of the prescribed dose at 110 cm.

Increased dose measured by film at the top of the profiles may result from scattered dose off the overhead vertex reflector. To test this hypothesis, a 3.5 cm thick piece of polystyrene was placed at the top of the phantom to cover the exposed Styrofoam (Figure 6). Films irradiated at 110, 160, 170, and 179 cm without the beam degrader were compared to both a film profile taken at day T = 523 and archived IC data (Figure 7). The maximum relative dose measured by film was reduced from +9.5% (without polystyrene) to +3.8% (with polystyrene), which was within the +4.7% dose reported for matched archived IC profiles. Thus, if the film backing is replaced with polystyrene, then film may be used to measure these profiles.

Overall, this work has established RCF as a comparable dosimeter to ICs; however, the RCF protocol is substantially more efficient. A total of two irradiations (1 per gantry angle) and 8 min was needed to irradiate all RCF for one profile; adding in preparation time resulted in a profile measurement time of 30 min. Alternatively, for IC measured profiles consisting of seven heights, a total of 56 irradiations, each of which took 24s to deliver 50cGy to d_ref_, was required. With substantial extra time required for gantry and polarity changes, each IC profile took approximately 3 h to measure. Overall, RCF provides an increase in time efficiency of 84%. With this time savings, the spatial resolution of the profiles and the frequency of QA may be increased.

## 4 CONCLUSIONS

In this work, we present an efficient protocol for measuring vertical profiles for TSET electron fields using RCF dosimetry. RCF profile measurements are stable over time and agree with IC dose measurements, making it a suitable dosimeter for TSET profiles. Adoption of film for these measurements at our center has already realized significant time savings. Ongoing developments will further improve the quality and reliability of these profiles. Future work includes the design, construction and commissioning of a new stand that incorporates a polystyrene backing for film measured profiles. Additionally, the effects of degrader thickness on profile uniformity should be studied and measuring continuous profiles using long strips of film will be implemented.

## AUTHOR CONTRIBUTIONS

Experimental Conception and Design: Martin G. Shim. Implementation: All authors. Analysis: All authors. Interpretation: All authors. Writing and revision of manuscript: All authors.

## CONFLICT OF INTEREST STATMENT

No conflicts of interest.

## DATA AVAILABILITY STATMENT

Data available on request from the authors.
